# Biosecurity measures for backyard poultry in developing countries: a systematic review

**DOI:** 10.1186/1746-6148-8-240

**Published:** 2012-12-07

**Authors:** Anne Conan, Flavie Luce Goutard, San Sorn, Sirenda Vong

**Affiliations:** 1 Epidemiology and Public Health Unit, Institut Pasteur du Cambodge, Réseau International des Instituts Pasteur, Phnom Penh, Cambodia; 2 Centre de coopération internationale en recherche agronomique pour le développement (CIRAD), Département ES, UR AGIRs, TA C22/E, Campus international de Baillarguet, Montpellier cedex 5, 34398, France; 3 National Veterinary Institute, Ministry of Agriculture, Forestry and Fisheries, Phnom Penh, Cambodia

**Keywords:** Biosecurity, Poultry, Backyard flocks, Scavenging, Infectious disease, H5N1 HPAI, Newcastle disease

## Abstract

**Background:**

Poultry represents an important sector in animal production, with backyard flocks representing a huge majority, especially in the developing countries. In these countries, villagers raise poultry to meet household food demands and as additional sources of incomes. Backyard production methods imply low biosecurity measures and high risk of infectious diseases, such as Newcastle disease or zoonosis such as Highly Pathogenic Avian Influenza (HPAI).

We reviewed literature on biosecurity practices for prevention of infectious diseases, and published recommendations for backyard poultry and assessed evidence of their impact and feasibility, particularly in developing countries. Documents were sourced from the Food and Agriculture Organization (FAO) website, and from Pubmed and Google databases.

**Results:**

A total of 62 peer-reviewed and non-referred documents were found, most of which were published recently (after 2004) and focused on HPAI/H5N1-related biosecurity measures (64%). Recommendations addressed measures for flock management, feed and water management, poultry trade and stock change, poultry health management and the risk to humans. Only one general guideline was found for backyard poultry-related biosecurity; the other documents were drawn up for specific developing settings and only engaged their authors (e.g. consultants). These national guidelines written by consultants generated recommendations regarding measures derived from the highest standards of commercial poultry production. Although biosecurity principles of isolation and containment are described in most documents, only a few documents were found on the impact of measures in family poultry settings and none gave any evidence of their feasibility and effectiveness for backyard poultry.

**Conclusions:**

Given the persistent threat posed by HPAI/H5N1 to humans in developing countries, our findings highlight the importance of encouraging applied research toward identifying sustained and adapted biosecurity measures for smallholder poultry flocks in low-income countries.

## Background

In 2009, the Food and Agriculture Organization of the United Nations (FAO) estimated the global population of domestic chickens and ducks at over 18 billion and 1 billion, respectively. Based on the number of animals, poultry represents the largest domestic animal stock in the world [1]. The industry is dominated by commercial farms while in developing countries, production consists of village or “backyard” (traditional) poultry, which is often extensive [2,3]. Backyard poultry is characterized by small flocks with low biosecurity measures. Backyard flocks represent around 80% of poultry stocks in many developing countries [3,4], often consisting of free indigenous unselected breeds of various ages, with various species mixed in the same flock [4-7]. Poultry closely interact with humans in the same household as well as with wild birds and other livestock where they are also exposed to vermin and predators. Poor or absent disease control strategies and inadequate management practices result in high levels of baseline mortality due to predators (e.g. rodents, snakes, small carnivores) or infectious diseases (e.g. Newcastle Disease (ND), salmonellosis, Gumboro disease or fowl typhoid) [2,8-12]. Backyard poultry raising usually requires low investments and death among poultry commonly occurs. As such poultry raising is often not the primary source of livelihood for backyard poultry farmers, nor is it the primary farming activity. However, it contributes significantly to incomes and home food consumption in rural areas of many developing countries [13,14]. In some settings or conditions, major losses of poultry flocks can result in malnutrition [15].

In several countries, poultry raising and consumption are also linked to socio-cultural factors such as religion or festivities [16-18], and to economic factors at farm and national levels [2,19,20]. Moreover some infectious poultry diseases are zoonotic, resulting in mild symptoms in humans (such as ND) [21], a range of mild to serious diseases (such as campylobacteriosis or psittacosis) [22,23] or can have fatal consequences in both poultry and humans, such as the Highly Pathogenic Avian Influenza (HPAI) A/H5N1 virus. Of these, some have raised potential public health concerns [24]. To avert human health risks and economic losses, biosecurity measures are implemented in farms to prevent the introduction, persistence or dissemination of infectious agents, through isolation, traffic control and/or sanitation measures. The rapid growth in intensive poultry production combined with increasing animal and human movement across the world is thought to have significantly contributed to the emergence of new pathogens (e.g. HPAI A/H5N1 or H9N2). However, in some settings there is evidence of sustained dissemination of these avian viruses between semi-extensive or backyard poultry flocks from area to area [25]. Inadequate backyard flock hygiene highlights the issue of poultry disease control in backyard systems [3,26]. In this context, we conducted a systematic literature review to analyse the evidence on the recommendations and use of biosecurity measures adapted to backyard poultry with a particular focus on developing countries.

## Methods

Recognizing the complexity of production systems and the fact that other terminologies have been used in various countries to describe backyard poultry depending on the differences in general husbandry and agricultural systems, we developed a protocol that consisted of the following items. Firstly, abiding by the PRISMA (Preferred Reporting Items for Systematic Reviews and Meta-Analyses) requirements, we performed a systematic literature search using the United States National Library of Medicine and the National Institutes of Health Medical Database (PubMed) and Google with no starting time limits, up until November 2011. As keywords we selected “poultry” associated with any of the following: “biosecurity”, “risk factors”, “knowledge”, “attitude” or “practice”. “Risk factors” documents were expected to provide recommendations on biosecurity measures against infectious diseases; we expected the studies on “Knowledge, Attitude and Practice” (KAP) to describe biosecurity practices, highlighting the needs for improvement.

Secondly, guided by several FAO staff members in the Livestock Production Systems Branch, we obtained additional unpublished documents and reports directly from the Agriculture department of the Animal Production and Health Division (AGAH) or their website devoted to avian influenza ( http://www.fao.org/avianflu/en/index.html).

**Figure 1:**
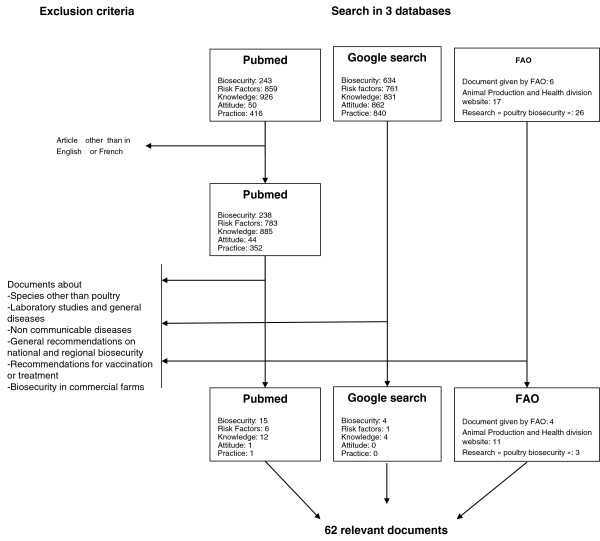
Flow diagram of studies selection process, by database and keyword, to obtain the 62 reviewed documents.

Finally, because the backyard poultry term is not universally used, we chose to inspect each identified article or report, including those that referred to backyard flocks and infectious agents or diseases. Our definition of backyard poultry encompassed (1) similar terms such as “indigenous poultry”, “native poultry”, “scavenging poultry”, “village poultry”, “local poultry”, “traditional poultry” or “free-range poultry” [5,27] and (2) small scale semi-intensive systems (e.g. ducks free grazing in rice fields) [3]. Backyard poultry is commonly associated with poor biosecurity conditions, small size (under 100 heads per flock) [2] and poultry raised by a family or in a household in rural or peri-urban areas. Parallel to the FAO classification (four sectors; 1: industrial ; 2 and 3: commercial; 4: backyard) [28], our search would include sectors 3 and 4. However, we purposely excluded from the review the small-scale intensive poultry system, because of its different management system; a system characterized by higher levels of biosecurity and overall husbandry conditions [6,7]. We also excluded laboratory studies, reviews about specific infectious diseases, studies that were not farm-based (all spatial studies and area, national or regional biosecurity studies were excluded), studies on vaccination or other treatments and those whose contents were not scientific (Figure 1). Relevant references found in the selected documents were also searched and reviewed. Our search was restricted to articles and reports written in English or French.

## Results

The literature search identified 62 different references relating to biosecurity issues in backyard flocks including 35 (56%) from PubMed, 18 (29%) from FAO reports and nine (15%) additional reports from Google search (Figure 1). However three documents (11%) - two on Google search [29,30] and five on PubMed [31] – that could not be obtained via the internet or our FAO contacts were dismissed from the review. The 59 remaining available documents referred to Europe (n = 1), Oceania (n = 2), America (n = 7), Asia (n = 23) and Africa (n = 20), including 47 for developing countries. The remaining documents did not refer to a particular area (n = 6). Field study articles (i.e. 6 case–control studies, 25 cross-sectional studies and 1 prospective longitudinal study) accounted for 54% (n = 32) of the relevant documents, followed by FAO reports (n = 12, 20%) and guidelines published by FAO or other organisations (n = 5, 8%). The ten other documents included descriptions of projects, reports, modelling or review articles, and PhD theses. Overall 23 documents (39%) provided precise biosecurity-related recommendations at flock level. Interestingly, there has been a surge in the number of reviewed documents since 2004; prior to this date, only four documents were found, dated 1998, 2000 and 2003. Avian influenza (n = 38, 64%) was the predominant subject of the documents we identified and selected. One study on ND-related risk factors provided no relevant results and was therefore not retained [32], reducing the total number of identified documents to 58.

The relevant documents were summarized and presented in Additional file 1: Annex 1 according to bibliographic information, research key (data base and keywords), geographic distribution, type of study, objectives given by authors, biosecurity-related findings and recommendations.

The FAO and OIE (World Organisation for Animal Health) define biosecurity as the implementation of measures to reduce the risk of the introduction and spread of disease agents [33,34]. Although ways of classifying these measures may vary, they all refer to the same basic principles of bioexclusion (i.e. preventing infectious agents from entering the farm) and biocontainment (i.e. preventing infectious agents from exiting) [35] and were implemented via: segregation to raise barriers to infectious diseases, cleaning and disinfection [33-35]. These two principles encompass the notions of (i) isolation, which ensures no contamination of flocks through housing and personal protection equipment; (ii) traffic control, which restricts the movement of products, stocks and persons; (iii) sanitation which includes methods for farmers to maintain disinfection and cleanliness in flocks. Specific recommendations for backyard poultry settings were found in ten FAO reports including eight for African countries and two for Asian countries – only engaging their authors – all of which referred to HPAI A/H5N1-related risks. There, the recommendations on biosecurity are listed by sector (described as sector 3 and sector 4) and/or type of recommendations [36-41] or otherwise [42-45].

No standardized classification exists to describe biosecurity measures. Based on the classification found in FAO documents, we present the result of this review using the following categories: flock management, feed and water, trade and stock, health management and risk to humans.

One recommended husbandry measure is to separate poultry by age and species or to consider raising one species instead of several [46], given that mixing species increases HPAIA/H5N1 virus transmission [47]. Age separation would facilitate the all in – all out strategy whereby sanitary cleaning can be carried out between the complete exit and renewal of flocks. However FAO recognizes that age separation would not be feasible in developing countries as most villagers raise free-ranging poultry for their own consumption. Firstly, the sale of all animals at a fixed age is not practiced by farmers, who keep several ages to ensure meat production throughout the year [34,41,48]. Secondly, farmers use their laying hens to renew the flocks [49,50], so the production of chicks can last throughout the year. Building fences to separate species and limit free-ranging is an important recommendation. On the one hand this measure is considered relatively affordable for farmers, as they can use local materials [46,51-54]; on the other hand fencing poultry increases the daily supplementary food requirements of the flock [6,14]. Indoor raising is also recommended, and actually often followed by US backyard poultry farmers [55]. However, recognizing the difficulties in applying this measure in developing countries [56,57], the authors nevertheless recommended at least keeping poultry indoors at night [8,58]. Interestingly, in many settings where the above housing recommendations are followed, farmers were motivated by non-biosecurity related incentives, e.g. confinement with fences implemented to prevent robbery, avoid dirt entering the house or losing birds [59]. Moreover, as shown by a study conducted in Nigeria, keeping flocks indoors without knowing the basic principles of biosecurity could actually expose humans to flocks, resulting in a higher risk of HPAI A/H5N1 infection [48]. Other husbandry measures involve cleaning and disinfecting the surroundings, which proves particularly effective in interrupting potential HPAI A/H5N1 [60]. Disinfection would also include materials, people (footwear, hand-washing) and buildings. All these measures are known and frequently used for North American backyard poultry [55,61,62] , while many farmers from developing countries may not know how to use disinfectants to protect their birds [48,51,52,58,63,64].

Many studies confirmed the potential risk of small backyard flocks roaming in or near waterlands and thus being exposed to Avian Influenza or ND virus-infected wild birds or contaminated environments [53,65,66]. The presence of ponds or canals was identified as increasing the risk of HPAI outbreaks in the village and the spread to neighbouring villages [60,67,68]. Again despite high awareness of greater risk of HPAI virus transmission from wild birds, contacts remain frequent between domestic and wild birds as observed in Egypt [63], the USA and New Zealand [55,62,65]. Authors recommended the use of bird pens to mitigate contact between domestic and wild birds [61]; however, no data were found on the feasibility and effectiveness of such a measure in HPAI A/H5N1 epidemic-prone countries.

Within husbandry practices, restricting people movement limits the risk of introducing infectious agents into flocks (e.g. HPAI) [47,48]. Some authors from countries where intensive farms are well-developed raised the issue of restricting contacts between commercial farms and backyard poultry [51,55,61,63]. This principle of visitor restriction appears to be well accepted among backyard poultry owners of developed or transitional countries [61,62]. Another FAO recommendation with respect to husbandry practices involves keeping a good record of flock history [38]. This animal observation allows the farmer to detect any changes in the flocks. Moreover, in the case of investigation, events would be easier to interpret if the flock history is known [8,53,61].

FAO recognizes food and water management as a biosecurity hazard to poultry, hence the need to account for it [37,39,40,43]. Consequently, recommendations include providing supplemented food (if possible) or ensuring clean containers for food and water [46,59]. However, there is evidence of an association between an untreated water source for poultry and outbreaks of HPAI A/H5N1 [68]. No practical solutions were proposed to address the latter hazards for backyard poultry, despite the need, as farmers from developing countries often use water from ponds or rivers for their poultry [53,65].

Health management includes the management of outbreaks and the use of litter. When there is an outbreak in the flock, sick birds should be separated as they may be a potential risk for the transmission of infectious disease [26,27,39,41,69]. The culling of sick animals by farmers was suggested as a radical measure in the US [61] but is hardly applicable in developing countries, where many farmers cannot afford to lose the entire flock. In view of the HPAI A/H5N1 threat, there is a strong, clearly-stated recommendation that dead birds be buried or burned [46,51,70]. The disposal of sick animals and carcasses is common practice in developed countries [61,65]. However, inappropriate implementation can increase the risk of ND infection [71] while many villagers in developing countries continue to sell sick or dead birds [14,72,73]. The use of untreated poultry manure as fertilizer poses a serious risk of infection spread [46]. This can be addressed by composting manure outside the flock area [46,61,65], a measure that is efficient but not well known among village farmers of many developing countries.

Poultry trading is often viewed as a risk factor for HPAI or ND in the flock and the village [60,67,68,71,74,75]. Backyard poultry farmers are therefore advised to avoid visiting live bird markets or other trading places [46,61]. However, this practice persists in many developing countries [8,48]. Poultry farmers are also advised to ensure that the poultry supply source is disease-free [37]. Ways of applying these recommendations were observed for instance in a study from Myanmar where farmers tend to purchase from a known and regular stock source such as their neighbours – provided the flock is disease free - rather than at live bird markets [14]. This principle is particularly well understood in developed countries where it is common practice in North America or New Zealand for backyard poultry owners to hatch their own eggs [65] or to buy chicks (or same age young adult birds) from one or a small number of the same commercial sources [55,61,62]. Because the risk of HPAI A/H5N1 transmission is actually higher when birds are brought in from another backyard flock [60], the subsequent related crucial recommendation is that newly introduced birds should be quarantined for two weeks before joining the flock to allow a time-lag for any disease to reveal itself [55,61].

Lastly, recommendations are made to limit the risk to humans. This includes separating children from poultry [52,54,59,63] and personal hygiene measures like hand washing or wearing gloves when handling poultry [55,63,76].

All the above recommendations are listed in different guidelines. Some were published by Agronomes et Vétérinaires Sans Frontières (AVSF) as recommendations for Cambodia, Vietnam and the Caribbean [33,77,78] or by DEFRA in the United Kingdom [79]. One of the articles also mentioned recommendations for avian influenza in all sectors [35] while two focused on backyard flocks [54,80]. These documents introduce the principles of biosecurity, and provide a list of the measures described above for application ranging from backyard flocks to veterinary paraprofessionals. A guide for professionals has been drawn up for ND [69]. Measures are also listed by Sharma *et al.*[81] giving the keys for developing biosecurity in Nepalese farms.

There are few publications (11) available that describe or analyse the impact of biosecurity measures on backyard flocks. Among these, three studies showed that information campaigns on flock management would improve the “general condition of the flock” [8,46,82]. Secondly, model-based evidence showed the positive economic effects of biosecurity in backyard poultry [68]. In articles about biosecurity, impact is confused with input or process indicators such as the number of trained people. Nevertheless, there is a general understanding that interventions require community participation and ownership to be successful [83,84]. Other papers on biosecurity input conclude that gender and age analyses should be included in husbandry and training study, because knowledge of HPAI A/H5N1 for example is correlated with socio-economic factors [85]. However, behaviour towards biosecurity varies according to other social factors (marital status for example), hence the inclusion of gender and economic strategy analysis to promote adequate intervention [70,86-88].

## Discussion

This review confirms the challenges of raising backyard poultry in such a way as to limit poultry deaths or morbidity due to well-known infectious agents, and discusses how to abide by biosecurity measures that are adapted and financially acceptable [54]. Although basic principles of biosecurity are undisputed regardless of poultry sector, few documents have been published about the impact and efficiency of biosecurity measures in backyard poultry flocks. As a result, guidelines on specific recommendations for improving biosecurity are limited. We found few FAO recommendations, most of which were written in the form of reports following specific country requests or consultancies that only engage their authors. Interestingly, the large majority of these documents have been issued since 2004, as they referred to or were requested following the pandemic threat posed by HPAI A/H5N1 virus infection in humans and birds. Most of these documents were funded by short term projects specifically geared towards emergency response to HPAI A/H5N1 instead of the willingness for government to invest in a long-term program. Even in the US and other developed countries, only fact sheets are produced by different organizations or universities [89,90]. There appear to be no national guidelines with practical information about biosecurity for backyard poultry [61].

Secondly, scientific articles looking at specific, adapted solutions to improve the control of infectious diseases in backyard poultry in developing countries are scarce. Despite the fact that 80% of the global poultry population is backyard-raised [3,4], some recommendations in the guidelines or reports are based on indirect evidence as to their efficiency and technical feasibility. However, a number of financial constraints were recognized in implementing these measures in a resource-poor setting. With the exception of one economic model [91], there are virtually no cost-benefit studies using field data. Guidelines have been issued to train farmers on how to reduce contacts with domestic birds and increase biosecurity in backyard flocks [33,69,77,78]. Studies showed that despite these training programmes and high awareness of transmission risk due to HPAI A/H5N1, a significant proportion of villagers continue their at-risk behaviours and practices; like in Egypt and elsewhere, many families rely on backyard poultry for their livelihood contributing to food security [64,76]. This discrepancy is likely explained by the fact that measures are often costly and may not be adapted to the economic considerations inherent to backyard poultry [54]. As reported many villagers tend to change their practices when these measures are economically beneficial [92,93]. Free ranging, for instance, is practised to enable easier and cheap access to feed on the ground or water from ponds or rivers. There is a paucity of data demonstrating the real impact of these measures [72]. Instead, we were only able to identify studies on the impact or effectiveness of these measures, evaluated according to the number of trained people [84] or the absence of outbreaks without control groups [82]. We are left with the impression that the proposed lists of recommendations were made without weighing biosecurity measures according to prioritization criteria, efficiency or financial and technical feasibility. Indeed, we believe that these control measures often derive from facts and evidence demonstrated in intensive sectors [94-96]. Compared with backyard poultry sector related studies, the number of studies from Pubmed on biosecurity in commercial farms is much higher (46 versus 15 for intensive and backyard respectively) (data not shown). As a major global industry, poultry mass production warrants the highest level of biosecurity to prevent the introduction and transmission of known pathogens. Resources have therefore been made available to optimize profits by identifying the most cost-effective measures using sound, robust methodologies such as cluster randomized and controlled trials [94].

Biosecurity implementation requires awareness, resources and the perception of higher risk and loss of profit. Unfortunately, as these conditions are not met, there is insufficient interest in the need to protect backyard poultry. This situation is likely related to multiple factors including a combination of the low economic importance of backyard poultry worldwide, absence of synergetic interests in zoonotic diseases between public health and livestock-related health, and the fact that backyard poultry is thought to pose little infectious disease-related risk to commercial farms [34,97]. Firstly, poultry rearing is often a secondary activity, a means of generating additional food high in protein content and nutritional value [27], and of generating additional income [26,92]. Implementation of basic biosecurity measures in villages to safeguard poultry is not seen as a priority. When there is low investment in poultry rearing, mortality is common, and is not seen as damaging for the household [13,14]. Secondly, prior to the occurrence of HPAI A/H5N1 epidemics and epizootics, most infectious diseases affecting backyard poultry were of little or no concern for public health. Many of these infections were non-zoonotic or involved mild infection in humans (e.g. ND, Fowl cholera) and outbreaks due mostly to salmonella or campylobacter from backyard poultry to humans rarely cause human death and often go undetected or under-recognized in developing countries [98,99]. Thirdly, it is thought that in developed countries commercial operations or farms that practice good biosecurity have fairly low transmission from backyard flocks [62]. In investigations conducted in North America or Europe backyard flocks appeared to have played little part in disease spread between commercial poultry farms [97,100,101]; hence the low investment for public health research on biosecurity adapted to backyard flocks throughout the world.

Although biosecurity is not a recent issue, the threat of HPAI A/H5N1 since 2004 to humans and poultry production (in terms of public health and economy) has underlined the lack of biosecurity in backyard farms in developing countries. Backyard flocks in high HPAI A/H5N1 virus transmission areas were initially thought as having a higher likelihood of HPAI A/H5N1 infection than commercial flocks because of higher frequency of exposure to wild birds [102-104]. Although spatio-temporal studies have proved the presence of free range duck flocks as a risk factor of HPAI A/H5N1 at regional level [105], recent studies indicate instead a lower risk of HPAI A/H5N1 in backyard flocks at farm level [106-109]. The current view is that no system is more to blame for infectious disease spread, and that biosecurity levels have to be increased in both commercial and backyard poultry systems [108,110]. In addition, the transmission of HPAI A/H5N1 is officially enzootic in 4 countries (Indonesia, Vietnam, Bangladesh, Egypt) according to FAO [111] and probably in other countries such as Cambodia [112]. Even drastic biosecurity in commercial poultry production systems in both the developed and developing worlds would hardly prevent the introduction of HPAI A/H5N1 or other infectious agents in the free-disease areas if biosecurity in the backyard sector does not increase dramatically. As shown in California, USA, exotic ND was transmitted to commercial farms in multiple geographic regions by bird and human movements associated with the backyard-flock sector [113].

Admittedly, our review may not be exhaustive and complete. We may well have missed unpublished observations and studies, particularly those conducted at a small scale or as part of a community development project. Indeed, many of the latter projects may have involved assessing biosecurity measures that improved livestock production in rural areas. Nevertheless, should these data exist, the appropriate evaluation of measures for backyard poultry settings is lacking and practical information is not readily available.

To date, control of HPAI A/H5N1 in endemic countries has basically relied on poultry vaccination (e.g. China, Indonesia, Egypt or Vietnam) and massive culling whenever HPAI A/H5N1 is detected [114,115]. However, these interventions have been difficult to sustain [116]. Furthermore, their efficiency in eliminating the virus from the poultry population is yet to be evidenced: vaccination as in Egypt [116] or culling with or without compensation policies as in Southeast Asia [117] can prove counterproductive i.e. economic losses that discourage reporting [118]; and new costs related to vaccination are not readily supported by backyard poultry farmers. In view of the mounting evidence that HPAI A/H5N1 can be transmitted through contaminated environments [119,120], we recommend that biosecurity measures, if appropriate, should be better promoted as a crucial intervention in containing H5N1 circulation. The international animal and public health community should encourage further research or projects to identify sustainable measures, which must be practical and proportionate to the risk [34,115]. In addition, we believe that the correct approach to zoonotic diseases should be holistic, based on the principle of improving personal and community hygiene to prevent all infectious diseases in backyard poultry to eventually mitigate exposure and transmission risk to humans. The keys to success and sustainability would undoubtedly involve engaging the community [34] and assessing the impact and economic benefit of a healthy livestock thanks to community hygiene.

## Conclusion

Our review confirmed that biosecurity is considered as an indispensable tool to mitigate the spread of infectious diseases. However, many recommendations for backyard flocks are not entirely practical. No general guidelines were found for backyard poultry-related biosecurity in developing countries. Although biosecurity principles of isolation and containment remain, few documents were found about the impact of measures in backyard settings and none gave any evidence of their feasibility and effectiveness. Moreover, most of the studies were short-term research and lacked evaluations of the sustainability of the recommended biosecurity measures. Long-term national programs should be envisaged in the future. Given the persistent threat posed by HPAI A/H5N1 to humans in developing countries, our findings highlight the importance of encouraging applied research toward identifying sustained and adapted biosecurity measures for backyard poultry flocks in low income settings.

## Abbreviations

AGAH: Animal production and health division; AVSF: Agronomes et veterinaires sans frontieres; FAO: Food and agriculture organization; HPAI: Highly pathogenic avian influenza; KAP: Knowledge attitude and practices; ND: Newcastle disease; OIE: World organization for animal health.

## Competing interests

The authors declare that they have no competing interests.

## Authors’ contributions

AC and SV designed the study. AC conducted the literature review and wrote the manuscript. FG, SS and SV were instrumental in collecting data and the grey literature and reviewed the manuscript. All authors approved the final version.

## Supplementary Material

Additional file 1 **Annex 1.** Main characteristics and information of studies included in the review after the study selection process. Click here for file

## References

[B1] FAO Faostat. Production. Live animals 2012 http://faostat.fao.org/site/573/default.aspx#ancor

[B2] Sonaiya EB Swan SEJ Small-scale poultry production 2004 Rome: FAO

[B3] Sonaiya F Smallholder family poultry as a tool to initiate rural development International Conference Poultry in the Twenty-first Century: avian influenza and beyond: 5–7 November 2007 2008 Bangkok, Thailand: FAO

[B4] Pym R Guerne Bleich E Hoffmann I The relative contribution of indigenous chicken breeds to poultry meat and egg production and consumption in the developing countries of Africa and Asia12th European Poultry conference: 10–14 September 2006 2006 Italy: World’s Poultry Science Assoc 197

[B5] Minga U Msoffe PL Gwakisa PS Biodiversity (variation) in disease resistance and in pathogens within rural chicken population22nd World’s Poultry Congress: June 8–12 2004 2004 World’s Poultry Science Assoc, Istanbul, Turkey

[B6] Singh DP Fotsa JC Opportunities of poultry breeding programmes for family production in developing countries: The bird for the poor E-conference of the International Network for Family Poultry Development 24 Jan-18 Feb 2011 2011 1 18

[B7] Gueye EF Gender aspects in family poultry management systems in developing countries Worlds Poult Sci J 2005 61 39 46 10.1079/WPS200440

[B8] Abdelqader A Wollny CB Gauly M Characterization of local chicken production systems and their potential under different levels of management practice in Jordan Trop Anim Health Prod 2007 39 3 155 164 10.1007/s11250-007-9000-x 17691540

[B9] Badubi SS Ravindran V Reid J A survey of small-scale broiler production systems in Botswana Trop Anim Health Prod 2004 36 8 823 834 15643818 10.1023/b:trop.0000045951.35345.17

[B10] Biswas PK Uddin GM Barua H Roy K Biswas D Ahad A Debnath NC Survivability and causes of loss of broody-hen chicks on smallholder households in Bangladesh Prev Vet Med 2008 83 3–4 260 271 17850905 10.1016/j.prevetmed.2007.08.001

[B11] Bell JG Factors limiting production efficiency and profitability from smallholder poultry production Worlds Poult Sci J 2009 65 207 210 10.1017/S0043933909000142

[B12] Ison AJ Spiegle SJ Morishita TY Predators of poultry 2012 http://ohioline.osu.edu/vme-fact/0022.html

[B13] Liao QY Lam WW Dang VT Jiang CQ Udomprasertgul V Fielding R What causes H5N1 avian influenza? Lay perceptions of H5N1 aetiology in South East and East Asia J Public Health 2009 31 4 573 581 10.1093/pubmed/fdp043 19423546

[B14] Henning J Khin A Hla T Meers J Husbandry and trade of indigenous chickens in Myanmar–results of a participatory rural appraisal in the Yangon and the Mandalay divisions Trop Anim Health Prod 2006 38 7–8 611 618 17265778 10.1007/s11250-006-4425-1

[B15] FAO Highly pathogenic avian influenza: a rapid assessment of socio-economic impact on vulnerable households in Egypt Prepared by Georgina Limon, Nicoline de Haan, Karin Schwabenbauer, Zahra S Ahmed and Jonathan Rushton AHBL - Promoting strategies for prevention and control of HPAI 2009 Rome: FAO

[B16] Wilson RT Poultry production and performance in the Federal Democratic Republic of Ethiopia World’s Poultry Sci J 2010 66 3 441 454 10.1017/S0043933910000528

[B17] Fournier T Coping with new food-related risks / Autour de la grippe aviaire au Viet Nam Anthropology of food 2009 http://aof.revues.org/index5303.html

[B18] Aklilu HA Udo HMJ Almekinders CJM Van der Zijpp AJ How resource poor households value and access poultry: Village poultry keeping in Tigray, Ethiopia Agr Syst 2008 96 1–3 175 183

[B19] Fasina FO Sirdar MM Bisschop SPR The financial cost implications of the highly pathogenic notifiable avian influenza H5N1 in Nigeria Onderstepoort Vet Res 2008 75 39 46 10.4102/ojvr.v75i1.86 18575062

[B20] McDermott JP Coleman PJ Randolph T Methods for assessing the impact of infectious diseases of livestock - their role in improving the control of Newcastle disease in Southern Africa SADC planning workshop on Newcastle disease control in village chickens Proceedings of an International Workshop, Maputo, Mozambique 6–9 March 2000 2000 ACT: Australian Center for International Agricultural Research Proceedings No. 103, Bruce 118 126

[B21] Acha PN Szyfres B Zoonoses and communicable diseases common to man and animals: Chlamydioses, rickettsioses, and viroses 2003 Washington DC, USA: Pan American Health Organization

[B22] Beeckman DS Vanrompay DC Zoonotic Chlamydophila psittaci infections from a clinical perspective Clin Microbiol Infect 2009 15 1 11 17 10.1111/j.1469-0691.2008.02669.x 19220335

[B23] Padungton P Kaneene JB Campylobacter spp in human, chickens, pigs and their antimicrobial resistance J Vet Med Sci 2003 65 2 161 170 10.1292/jvms.65.161 12655109

[B24] WHO Influenza at the Human-Animal Interface. Summary and assessment as of 5 December 2011 2011 http://www.who.int/influenza/human_animal_interface/avian_influenza/Influenza_Summary_IRA_HA_interface.pdf

[B25] Loth L Gilbert M Wu J Czarnecki C Hidayat M Xiao X Identifying risk factors of highly pathogenic avian influenza (H5N1 subtype) in Indonesia Prev Vet Med 2011 102 1 50 58 10.1016/j.prevetmed.2011.06.006 21813198 PMC4868053

[B26] Permin A Detmer A Improvement of management and biosecurity practices in smallholder poultry producers 2007 FAO, Rome

[B27] Ahlers C Alders R Bagnol B Cambaza AZ Harun M Mgomezulu R Msami H Pym B Wegener P Wethli E Young M Improving village chicken production: a manual for field workers and trainers 2009 Australian Centre for International Agricultural Research (ACIAR), Bruce, ACT

[B28] FAO Production Systems characteristics http://www.fao.org/docs/eims/upload/214190/ProductionSystemsCharacteristics.pdf

[B29] Kohlhagen K Analysis and evaluation of the effectiveness of a poultry biosecurity and disease prevention curriculum 2008 West Lafayette: Purdue University

[B30] CARE international in Vietnam Knowledge, attitudes, practices study of small holder poultry raising farmers in response to Avian Influenzahttp://www.comminit.com/natural-resource/node/219322

[B31] Olugbenga-Bello AI Bamidele JO Oladele EA Ifeka JO Knowledge and practices of poultry workers on prevention of avian flu in Osogbo, Osun State, Nigeria Niger Postgrad Med J 2009 16 1 8 13 19305431

[B32] Otim MO Kabagambe EK Mukiibi GM Christensen H Bisgaard M A study of risk factors associated with Newcastle disease epidemics in village free-range chickens in Uganda Trop Anim Health Prod 2007 39 1 27 35 10.1007/s11250-006-4441-1 17941485

[B33] FAO, AVSF, DAH Prevention and control of Avian flu in small scale poultry. A guide for veterinary paraprofessionals in Vietnam 2005 Rome: FAO, AVSF, DAH

[B34] FAO Biosecurity for highly pathogenic avian influenza: issues and options 2008 Rome: FAO

[B35] Charisis N Avian influenza biosecurity: a key for animal and human protection Vet Ital 2008 44 4 657 669 20411493

[B36] Bebay C Biosécurité dans les élevages avicoles à petite échelle. Analyse et conditions d'amélioration au Cameroun et au Togo 2006 Rome: ECTAD/AGAP, FAO

[B37] Nyaga P Poultry Sector Analysis: Biosecurity Review and Improved poultry husbandry systems for sectors 3 and 4 to prevent HPAI infection in Uganda 2009 Rome: FAO

[B38] Nyaga P Good biosecurity practices in small scale commercial and scavenging production systems in Kenya 2007 Rome: FAO

[B39] Pagani P Kilany W Interventions for improving bio-security of small-scale poultry producers in Egypt 2007 Rome: FAO

[B40] Njue S Appropriate biosecurity practices for countering HPAI infection in sector 3 and 4 poultry production systems in selected areas of Kenya [in press] 2009 Rome: FAO

[B41] Msami H Good biosecurity practices in non integrated commercial and in scavenging production systems in Tanzania 2008 Rome: FAO

[B42] Dolberg F GuerneBleich E McLeod A Emergency Regional Support for Post-Avian Influenza Rehabilitation 2005 Rome: FAO

[B43] Pagani P Abimiku Y Emeka-Okolie W Assessment of the Nigerian poultry market chain to improve biosecurity 2008 Rome: FAO

[B44] Pagani P Wossene A Review of the new features of the Ethiopian poultry sector. Biosecurity implications 2008 FAO, Rome

[B45] VSF-CICDA Review of free-range duck farming systems in Northern Vietnam and assessment of their implication in the spreading of the Highly Pathogenic (H5N1) strain of Avian Influenza (HPAI) 2006 Rome: FAO

[B46] Cristalli A Capua I Practical problems in controlling H5N1 high pathogenicity avian influenza at village level in Vietnam and introduction of biosecurity measures Avian Dis 2007 51 1 Suppl 461 462 17494607 10.1637/7564-033106R.1

[B47] Henning KA Henning J Morton J Long NT Ha NT Meers J Farm- and flock-level risk factors associated with Highly Pathogenic Avian Influenza outbreaks on small holder duck and chicken farms in the Mekong Delta of Viet Nam Prev Vet Med 2009 91 2–4 179 188 19581011 10.1016/j.prevetmed.2009.05.027

[B48] Alhaji NB Odetokun IA Assessment of biosecurity measures against Highly Pathogenic Avian Influenza risks in small-scale commercial farms and free-range poultry flocks in the Northcentral Nigeria Transbound Emerg Dis 2011 58 2 157 161 10.1111/j.1865-1682.2010.01195.x 21205255

[B49] Olwande PO Ogara WO Okuthe SO Muchemi G Okoth E Odindo MO Adhiambo RF Assessing the productivity of indigenous chickens in an extensive management system in southern Nyanza, Kenya Trop Anim Health Prod 2010 42 2 283 288 10.1007/s11250-009-9418-4 19680773

[B50] Mopate L Lony M Survey on family chicken farms in the rural area of N’Djaména, Chad Livest Res Rural Dev 1999 11 2 http://www.lrrd.org/lrrd11/2/chad112.htm

[B51] Hamilton-West C Rojas H Pinto J Orozco J Herve-Claude LP Urcelay S Characterization of backyard poultry production systems and disease risk in the central zone of Chile Res Vet Sci 2012 93 1 121 124 10.1016/j.rvsc.2011.06.015 21752410

[B52] Datta S Sen S Sengupta B A study on knowledge and practice related to bird flu in a rural community of Hooghly District of West Bengal Indian J Public Health 2010 54 4 216 218 10.4103/0019-557X.77265 21372372

[B53] Jansen T Glatz PC Miao ZH A survey of village poultry production in the Solomon Islands Trop Anim Health Prod 2009 41 7 1363 1370 10.1007/s11250-009-9323-x 19242817

[B54] Aini I Biosecurity in family flocks Proceedings of the 21st World's Poultry Congress: 20–24 August 2000 2000 World’s Poultry Science Assoc, Montreal, Canada 49 54

[B55] Yendell SJ Rubinoff I Lauer DC Bender JB Scheftel JM Antibody prevalence of low-pathogenicity avian influenza and evaluation of management practices in Minnesota backyard poultry flocks Zoonoses Public Health 2012 59 2 139 143 10.1111/j.1863-2378.2011.01427.x 21824379

[B56] Barennes H Martinez-Aussel B Vongprahrachanh P Strobel M Avian Influenza risk perceptions, Laos Emerg Infect Dis 2007 13 7 1126 1128 10.3201/eid1307.061197 18214204 PMC2878220

[B57] Thekisoe MM Mbati PA Bisschop SP Diseases of free-ranging chickens in the Qwa-Qwa District of the northeastern Free State province of South Africa J S Afr Vet Assoc 2003 74 1 14 16 12836740 10.4102/jsava.v74i1.490

[B58] Lukman D Ridwan Y Wibowo B Basri C Sudarnika E Sugama A Hermans P Nell A Biosecurity practices in village poultry in Cipunagara Subdistrict, Subang District, West Java: Case study Proceedings of 1st International Congress of South East Asia Veterinary School Association: 20-22July 2010 2011 2011 Bogor, Indonesia: SEAVSA

[B59] Harvey SA Winch PJ Leontsini E Torres Gayoso C Lopez Romero S Gilman RH Oberhelman RA Domestic poultry-raising practices in a Peruvian shantytown: implications for control of Campylobacter jejuni-associated diarrhea Acta Trop 2003 86 1 41 54 10.1016/S0001-706X(03)00006-8 12711102

[B60] Paul M Wongnarkpet S Gasqui P Poolkhet C Thongratsakul S Ducrot C Roger F Risk factors for highly pathogenic avian influenza (HPAI) H5N1 infection in backyard chicken farms, Thailand Acta Trop 2011 118 3 209 216 10.1016/j.actatropica.2011.03.009 21459074

[B61] Burns TE Kelton D Ribble C Stephen C Preliminary investigation of bird and human movements and disease-management practices in noncommercial poultry flocks in southwestern British Columbia Avian Dis 2011 55 3 350 357 10.1637/9646-010411-Reg.1 22017030

[B62] Garber L Hill G Rodriguez J Gregory G Voelker L Non-commercial poultry industries: surveys of backyard and gamefowl breeder flocks in the United States Prev Vet Med 2007 80 2–3 120 128 17337307 10.1016/j.prevetmed.2007.01.012

[B63] Ismail NA Ahmed HA Knowledge, attitudes and practices related to Avian Influenza among a rural community in Egypt J Egypt Public Health Assoc 2010 85 1–2 73 96 21073849

[B64] Fatiregun AA Saani MM Knowledge, attitudes and compliance of poultry workers with preventive measures for avian influenza in Lagelu, Oyo State, Nigeria J Infect Dev Ctries 2008 2 2 130 134 10.3855/T2.2.130 19738338

[B65] Zheng T Adlam B Rawdon TG Stanislawek WL Cork SC Hope V Buddle BM Grimwood K Baker MG O'Keefe JS Huang QS A cross-sectional survey of influenza A infection and management practices in small rural backyard poultry flocks in New Zealand N Z Vet J 2010 58 2 74 80 10.1080/00480169.2010.65086 20383241

[B66] Awan MA Otte MJ James AD The epidemiology of Newcastle disease in rural poultry: a review Avian Pathol 1994 23 3 405 423 10.1080/03079459408419012 18671109

[B67] Desvaux S Grosbois V Pham TT Fenwick S Tollis S Pham NH Tran A Roger F Risk factors of highly pathogenic avian influenza H5N1 occurrence at the village and farm levels in the red river delta region in Vietnam Transbound Emerg Dis 2011 58 6 492 502 10.1111/j.1865-1682.2011.01227.x 21545692

[B68] Fasina FO Rivas AL Bisschop SP Stegeman AJ Hernandez JA Identification of risk factors associated with highly pathogenic avian influenza H5N1 virus infection in poultry farms, in Nigeria during the epidemic of 2006–2007 Prev Vet Med 2011 98 2–3 204 208 21146235 10.1016/j.prevetmed.2010.11.007

[B69] Alders R Spradbrow P Controling Newcastle Disease in village chickens. A field manual 2001 Australian Centre for International Agriculture Research (ACIAR), Bruce, ACT

[B70] Yakhshilikov Y Tiongco M Narrod C Friedman J Knowledge and practices of Indonesian rural communities and poultry farmers toward avian flu HPAI Research Brief 2009 17 9

[B71] Njagi LW Nyaga PN Mbuthia PG Bebora LC Michieka JN Minga UM retrospective study of factors associated with Newcastle disease outbreaks in village indigenous chickens Bull Anim Health Prod Afr 2010 58 1 22 33

[B72] Ly S Van Kerkhove MD Holl D Froehlich Y Vong S Interaction between humans and poultry, rural Cambodia Emerg Infect Dis 2007 13 1 130 132 10.3201/eid1301.061014 17370527 PMC2725837

[B73] Musa OI Aderibigbe SA Salaudeen GA Oluwole FA Samuel SO Community awareness of bird flu and the practice of backyard poultry in a North-Central state of Nigeria J Prev Med Hyg 2010 51 4 146 151 21553559

[B74] Biswas PK Christensen JP Ahmed SS Das A Rahman MH Barua H Giasuddin M Hannan AS Habib MA Debnath NC Risk for infection with highly pathogenic avian influenza virus (H5N1) in backyard chickens, Bangladesh Emerg Infect Dis 2009 15 12 1931 1936 10.3201/eid1512.090643 19961672 PMC3044532

[B75] Santhia K Ramy A Jayaningsih P Samaan G Putra AA Dibia N Sulaimin C Joni G Leung CY Sriyal J Peiris M Wandra T Kandun N Avian influenza A H5N1 infections in Bali Province, Indonesia: a behavioral, virological and seroepidemiological study Influenza Other Respi Viruses 2009 3 3 81 89 10.1111/j.1750-2659.2009.00069.x 19459276 PMC4634692

[B76] Olsen SJ Laosiritaworn Y Pattanasin S Prapasiri P Dowell SF Poultry-handling practices during avian influenza outbreak, Thailand Emerg Infect Dis 2005 11 10 1601 1603 10.3201/eid1110.041267 16318704 PMC3366731

[B77] FAO Guide for the prevention and control of avian flu in small scale poultry 2006 2 Rome: Regional Office for Latin America and the Carribean

[B78] FAO, VSF-CICDA Prevention and control of avian flu in small scale poultry. A guide for veterinary paraprofessionals in Cambodia Romes: FAO 1 36

[B79] DEFRA Biosecurity and preventing disease 2012 DEFRA http://www.defra.gov.uk/publications/files/pb11380-biosecurity-preventingdisease-060313.pdf

[B80] Grunkemeyer VL Zoonoses, public health, and the backyard poultry flock Vet Clin North Am Exot Anim Pract 2011 14 3 477 490 10.1016/j.cvex.2011.05.010 21872783

[B81] Sharma B Poultry production, management and bio-security measures J Agric Envir 2010 11 120 125

[B82] Bhandari DP Wollen TS Lohani MN Preventing highly pathogenic avian influenza (HPAI) at the rural community level: a case study from Cambodia Trop Anim Health Prod 2011 43 6 1071 1073 10.1007/s11250-011-9828-y 21442155

[B83] Sodjinou E Poultry-based intervention as tool for poverty reduction and gender empowerment: emperical evidence from Benin PhD Thesis 2011 Copenhagen: University of Copenhagen

[B84] Msoffe PL Bunn D Muhairwa AP Mtambo MM Mwamhehe H Msago A Mlozi MR Cardona CJ Implementing poultry vaccination and biosecurity at the village level in Tanzania: a social strategy to promote health in free-range poultry populations Trop Anim Health Prod 2010 42 2 253 263 10.1007/s11250-009-9414-8 19688307 PMC2809980

[B85] Leslie T Billaud J Mofleh J Mustafa L Yingst S Knowledge, attitudes, and practices regarding avian influenza (H5N1), Afghanistan Emerg Infect Dis 2008 14 9 1459 1461 10.3201/eid1409.071382 18760020 PMC2603107

[B86] Bagnol B Gender issues in small-scale family poultry production: experiences with Newcatle Disease and Highly Pathogenic Avian Influenza control World’s Poultry Sci J 2009 65 2 231 240 10.1017/S0043933909000191

[B87] Guerne Bleich E Pagani P Honhold N Progess towards practical options for improving biosecurity of small scale poultry producers World’s Poultry Sci J 2009 65 2 211 216 10.1017/S0043933909000154

[B88] Sen S Shane SM Scholl DT Hugh-Jones ME Gillespie JM Evaluation of alternative strategies to prevent Newcastle disease in Cambodia Prev Vet Med 1998 35 4 283 295 10.1016/S0167-5877(98)00065-8 9689660

[B89] Ebako GM Morishita TY Preventive Medicine for Backyard Chickens 2012 Ohio State University Extension Fact Sheet http://ohioline.osu.edu/vme-fact/0011.html

[B90] DEFRA Biosecurity guidance to prevent the spread of animal diseases 2012 London: DEFRA

[B91] Fasina FO Ali AM Yilma JM Thieme O Ankers P The cost-benefit of biosecurity measures on infectious diseases in the Egyptian household poultry Prev Vet Med 2012 103 2–3 178 191 21982688 10.1016/j.prevetmed.2011.09.016

[B92] Sultana R Rimi NA Azad S Islam MS Khan MS Gurley ES Nahar N Luby SP Bangladeshi backyard poultry raisers’ perceptions and practices related to zoonotic transmission of avian influenza J Infect Dev Ctries 2012 6 2 156 165 22337845 10.3855/jidc.2242

[B93] Wiegers E Curry J Understanding smallholder’s decisions towards adopting HPAI Prevention control measures HPAI Research Brief 2009 16 4

[B94] Gibbens JC Pascoe SJ Evans SJ Davies RH Sayers AR A trial of biosecurity as a means to control Campylobacter infection of broiler chickens Prev Vet Med 2001 48 2 85 99 10.1016/S0167-5877(00)00189-6 11154782

[B95] Halvorson DA Biosecurity on a multiple-age egg production complex: a 15-year experience Avian Dis 2011 55 1 139 142 10.1637/9580-101710-Case.1 21500651

[B96] Alexander DJ The epidemiology and control of avian influenza and newcastle disease J Comp Path 1995 112 2 105 126 10.1016/S0021-9975(05)80054-4 7769142

[B97] Bavinck V Bouma A van Boven M Bos ME Stassen E Stegeman JA The role of backyard poultry flocks in the epidemic of highly pathogenic avian influenza virus (H7N7) in the Netherlands in 2003 Prev Vet Med 2009 88 4 247 254 10.1016/j.prevetmed.2008.10.007 19178969

[B98] WHO Campylobacter Volume Fact sheet N°255 2011

[B99] Cardinale E Tall F Gueye EF Cisse M Salvat G Risk factors for Salmonella enterica subsp. enterica infection in senegalese broiler-chicken flocks Prev Vet Med 2004 63 3–4 151 161 15158567 10.1016/j.prevetmed.2004.03.002

[B100] Canadian Food Inspection Agency Low W, Chown L Comprehensive report on the 2004 outbreak of high pathogenicity avian influenza (H7N3) in the Fraser Valley of British Columbia, Canada Comprehensive report on the 2004 outbreak of high pathogenicity avian influenza (H7N3) in the Fraser Valley of British Columbia, Canada 2004 Ottawa, Canada: Canadian Food Inspection Agency

[B101] Akey BL Low-pathogenicity H7N2 avian influenza outbreak in Virginia during 2002 Avian Dis 2003 47 3 Suppl 1099 1103 14575120 10.1637/0005-2086-47.s3.1099

[B102] Werner O Starick E Grund CH Isolation and characterization of a low-pathogenicity H7N7 influenza virus from a turkey in a small mixed free-range poultry flock in Germany Avian Dis 2003 47 3 suppl 1104 1106 14575121 10.1637/0005-2086-47.s3.1104

[B103] Koch G Elbers AR Outdoor ranging of poultry: a major risk factor for the introduction and development of High-Pathogenicity Avian Influenza NJAS 2006 54 2 179 194

[B104] Terregino C De Nardi R Vubertu V Scremin M Reaffini E Martin AM Cattoli G Bonfanti L Capua I Active surveillance for avian influenza viruses in wild birds and backyard flocks in Northern Italy during 2004 to 2006 Avian Pathol 2007 36 4 337 344 10.1080/03079450701488345 17620182

[B105] Gilbert M Xiao X Chaitaweesub P Kalpravidh W Premashthira S Boles S Slingenbergh J Avian influenza, domestic ducks and rice agriculture in Thailand Agric Ecosyst Environ 2007 119 409 415 10.1016/j.agee.2006.09.001 18418464 PMC2311503

[B106] Otte J Pfeiffer D Tiensin T Price L Silbergeld E Evidence-based Policy for Controlling HPAI in Poultry: Bio-security revisited Pro-Poor Livestock Policy Initiative A living from livestock Research Report 2006 Baltimore: John Hopkins Bloomberg school of Public Health 13

[B107] Otte J Pfeiffer D Soares-Magalhaes R Burgos S Roland-Holst D Flock Size and HPAI Risk in Cambodia, Thailand and Vietnam HPAI Research Brief 2008 5 1 4

[B108] Walker P Cauchemez S Hartemink N Tiensin T Ghani AC Outbreaks of H5N1 in poultry in Thailand: the relative role of poultry production types in sustaining transmission and the impact of active surveillance in control J R Soc Interface 2012 9 73 1836 1845 10.1098/rsif.2012.0022 22356818 PMC3385766

[B109] Otte J Pfeiffer D Tiensin T Price L Silbergeld E Highly pathogenic avian influenza risk, biosecurity and smallholder adversity Livest Res Rural Dev 2007 19 7 102

[B110] Sims L Risks associated with poultry production systems International conference poultry in the twenty-first century 2008 Bangkok, Thailand: Avian Influenza and beyond: 5–7 November 2008 1 24

[B111] FAO H5N1 HPAI worldwide in 2010 Empres Transboundary Animal Diseases Bulletin 2011 37 21 29

[B112] Buchy P Fourment M Mardy S Sorn S Holl D Ly S Vong S Enouf V Peiris JS van der Werf S Molecular epidemiology of clade 1 influenza A viruses (H5N1), southern Indochina peninsula, 2004–2007 Emerg Infect Dis 2009 15 10 1641 1644 10.3201/eid1510.090115 19861062 PMC2866389

[B113] Breimeyer R Whiteford A Shere J California experience with exotic Newcastle disease: a state and federal regulatory perspective107th Annual Meeting of the United States Health Association: 2003 2003 San Diego, California: Pat Campbell and Associates, Richmond, VA 65 70

[B114] OIE Technical disease cards: Highly Pathogenic Avian Influenza http://www.oie.int/fileadmin/Home/eng/Animal_Health_in_the_World/docs/pdf/AVIAN_INFLUENZA_FINAL.pdf

[B115] Sims L Animal intervention strategies under different epidemiological and field condition that can reduce risk of zoonotic infection OFFLU review paper 2011 Paris, Rome: OIE, FAO

[B116] Cattoli G Fusaro A Monne I Coven F Joannis T El-Hamid HS Hussein AA Cornelius C Amarin NM Mancin M Holmes EC Capua I Evidence for differing evolutionary dynamics of A/H5N1 viruses among countries applying or not applying avian influenza vaccination in poultry Vaccine 2011 29 50 9368 9375 10.1016/j.vaccine.2011.09.127 22001877

[B117] Kanamori S Jimba M Compensation for Avian Influenza cleanup Emerg Infect Dis 2007 13 2 341 342 10.3201/eid1302.061391 17479909 PMC2725861

[B118] Hang V The effects of avian influenza on rural poultry farmers' livelihood. A case in Yen Son and Tan Binh communes - Tam Diep town - Ninh Binh province, Vietnam Master thesis 2010 Hanoi: National Institute of Animal Husbanry

[B119] Vong S Ly S Mardy S Holl D Buchy P Environmental contamination during influenza A virus (H5N1) outbreaks, Cambodia, 2006 Emerg Infect Dis 2008 14 8 1303 1305 10.3201/eid1408.070912 18680663 PMC2600401

[B120] Vong S Ly S Van Kerkhove MD Achenbach J Holl D Buchy P Sorn S Seng H Uyeki TM Sok T Katz JM Risk factors associated with subclinical human infection with Avian Influenza A (H5N1) virus–Cambodia, 2006 J Infect Dis 2009 199 12 1744 1752 10.1086/599208 19416078

